# A streamlined method for systematic, high resolution *in situ *analysis of mRNA distribution in plants

**DOI:** 10.1186/1746-4811-1-8

**Published:** 2005-10-06

**Authors:** Sinéad Drea, Julia Corsar, Brian Crawford, Peter Shaw, Liam Dolan, John H Doonan

**Affiliations:** 1 John Innes Centre, Norwich NR4 7UH, UK; 2 Department of Molecular, Cellular and Developmental Biology, P.O. Box 208104, Yale University, 266 Whitney Ave., New Haven, CT 06520-8104, USA

## Abstract

**Background:**

*In situ *hybridisation can provide cellular, and in some cases sub-cellular, resolution of mRNA levels within multicellular organisms and is widely used to provide spatial and temporal information on gene expression. However, standard protocols are complex and laborious to implement, restricting analysis to one or a few genes at any one time. Whole-mount and reverse transcriptase-PCR (RT-PCR) based protocols increase throughput, but can compromise both specificity and resolution. With the advent of genome-wide analysis of gene expression, there is an urgent need to develop high-throughput *in situ *methods that also provide high resolution.

**Results:**

Here we describe the development of a method for performing high-throughput *in situ *hybridisations that retains both the high resolution and the specificity of the best manual versions. This refined semi-automated protocol has the potential for determining the spatial and temporal expression patterns of hundreds of genes in parallel on a variety of tissues. We show how tissue sections can be organized on microscope slides in a manner that allows the screening of multiple probes on each slide. Slide handling, hybridisation and processing steps have been streamlined providing a capacity of at least 200 probes per week (depending on the tissue type). The technique can be applied easily to different species and tissue types, and we illustrate this with wheat seed and *Arabidopsis *floral meristems, siliques and seedlings.

**Conclusion:**

The approach has the high specificity and high resolution of previous *in situ *methods while allowing for the analysis of several genes expression patterns in parallel. This method has the potential to provide an analysis of gene expression patterns at the genome level.

## Background

*In situ *hybridisation (ISH) is the method of choice for describing the spatial expression pattern of a given gene. High resolution protocols provide cellular and even subcellular resolution. In multicellular organisms, ISH complements northern blotting, RT-PCR and microarrays, where the extraction of RNA from whole tissues invariably results in the loss of spatial information. Microarrays allow many genes to be studied in parallel and are currently one of the most powerful tools to study gene expression. However, microarray outputs often need to be verified by independent methods, such as ISH [1,2], and because these downstream methods have a much lower capacity, verification is usually limited to one or a few genes. ISH must therefore be made more efficient and less time-consuming.

A number of variations on the traditional *in situ *protocols have been reported, including whole-mount ISH (WISH) [3], *in situ *PCR [4,5] and the use of vibratome sectioned tissues [6]. The main shortcoming of ISH is undoubtedly the low-throughput nature of the technique. *In situ *PCR (ISPCR) and RT-ISPCR are elegant techniques that can increase both sensitivity and throughput but they are at best only semi-quantitative [5] and it is desirable first to ascertain the expression pattern by conventional means in order to establish suitable conditions for each probe.

Efforts to make the ISH technique into a highly parallel, systematic process have been successful in flies and primitive chordates [7-9]. Attempts have been made to address this issue in plants using WISH and *in situ *PCR techniques [10,11] although actual throughput remains undetermined.

High-throughput protocols used for animal embryos normally involve whole-mount methods [7,8,12], thus avoiding the need to section material. The challenges in applying similar techniques to plants include the large size of the tissues and the variable nature of the cell wall. These factors can variably compromise the penetration of probe and make microscopic examination more difficult and time-consuming. WISH is a possibility for *Arabidopsis *roots and seedlings [11], at least for low- and medium-throughput. However, when performed on other larger tissues, such as seeds, WISH may require embedding and sectioning after the *in situ *has been performed to evaluate the results [13]. Therefore, the high-throughput advantages gained in the early stages of such procedures are effectively cancelled out.

Promoter fusions with reporter genes are another option for cellular localisation of transcripts but this approach has recognised shortcomings [14]. Elements controlling gene expression are known to be located not only in the traditional promoter region upstream of the coding region, but intergenically and, potentially, a considerable distance from the gene [15,16]. The resources required for mass transformation and the fact that not all plant species are amenable limits the application of this approach to well-studied model species.

As well as providing an independent means of screening genes for the desired expression profiles (differential expression, domain specific expression etc.) in gene discovery efforts, we envisage that high-throughput mRNA ISH is entirely feasible and will complement the ever-growing microarray data resources available [2,29]; . Recently real-time RT-PCR has been adapted for high-throughput processing [30]. While these approaches provide a wealth of expression data for functional genomics, they are unable to provide the spatial resolution that often directly reflects functional involvement in developmental processes. This inherent drawback in microarray technology has been elegantly addressed using cell sorting to isolate pure populations of a given cell type from the *Arabidopsis *root. However, this innovative approach is limited to species where suitable and diverse cell line markers are available [31]. It is further limited to tissues whose cells can be separated and sorted: roots are susceptible to protoplasting enzymes but shoots and many other tissues are not.

With these considerations in mind, we have deconstructed the "traditional" ISH protocol and developed a protocol for ISH that retains high resolution and specificity but integrates a degree of automation to a standardised and streamlined protocol. We have used wheat grain and *Arabidopsis *floral meristems as tests for this new high-throughput protocol and show that it is capable of highly parallel processing.

## Results and discussion

### Generating an integrated protocol

**Figure 1:**
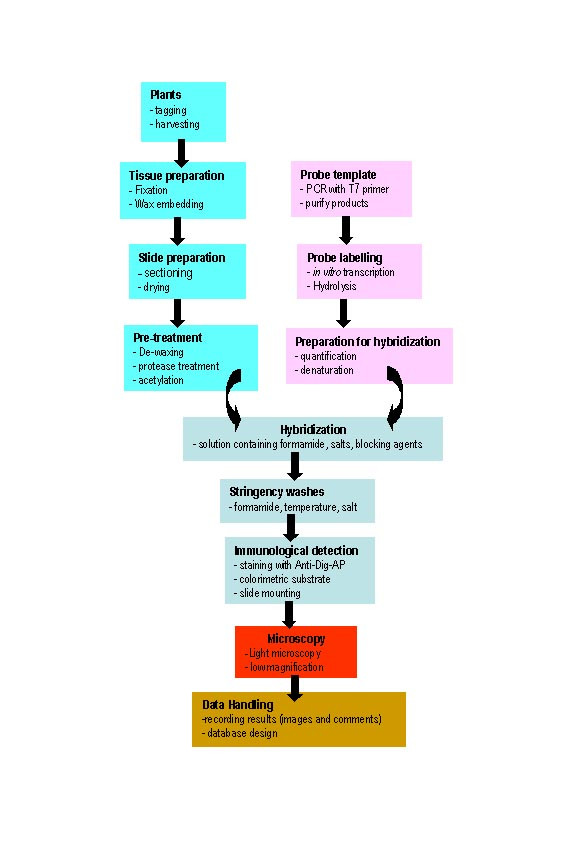
Flowchart summarising mRNA *in situ *methodology.

One of the main challenges in mRNA ISH is developing an economical protocol that is applicable to large batches of different probes while maintaining a high level of specificity, sensitivity and resolution. The level of economy must be maintained throughout the process to provide a systematic high-throughput level of work. Figure 1 summarises the ISH protocol as described in previous reports [17,18] and describes the five main components of the entire procedure. We have examined each of these components individually, but in context of the overall technique, with the view to (i) simplification, (ii) automation and (iii) optimisation. As a practical accompaniment to the following description we have included a step-by-step version of the protocol as used at the bench (Additional file 1).

#### (i) Plant tissue preparation

Plants grown under desired conditions were harvested, trimmed to allow penetration of solutions, and immediately fixed. These steps were carried out manually in both the standard and new protocols but, in order to automate the new protocol, the samples were placed in a Tissue-Tek Vacuum infiltration processor for further processing.

The Tissue-Tek machine permits use of a combination of vacuum and pressure to exchange solutions at defined times and temperatures, relieving the operator of a large number of tedious steps as well as standardising the process. The initial fixation, dehydration and wax infiltration steps can take over a week to complete using the manual protocol, whereas the automated protocol reduces this to 24 hours. We evaluated various plant tissues prepared by both methods for tissue integrity and preservation of mRNA (signal strength). *Arabidopsis *tissues are equally well preserved and stained by both protocols but the automated procedure provided enhanced preservation in developing wheat seeds (data not shown). Many plant tissues, such as mature leaves, are very difficult to embed in wax using the manual protocol but even these recalcitrant tissues can be accommodated by altering the timing or pressure of processing.

**Figure 2:**
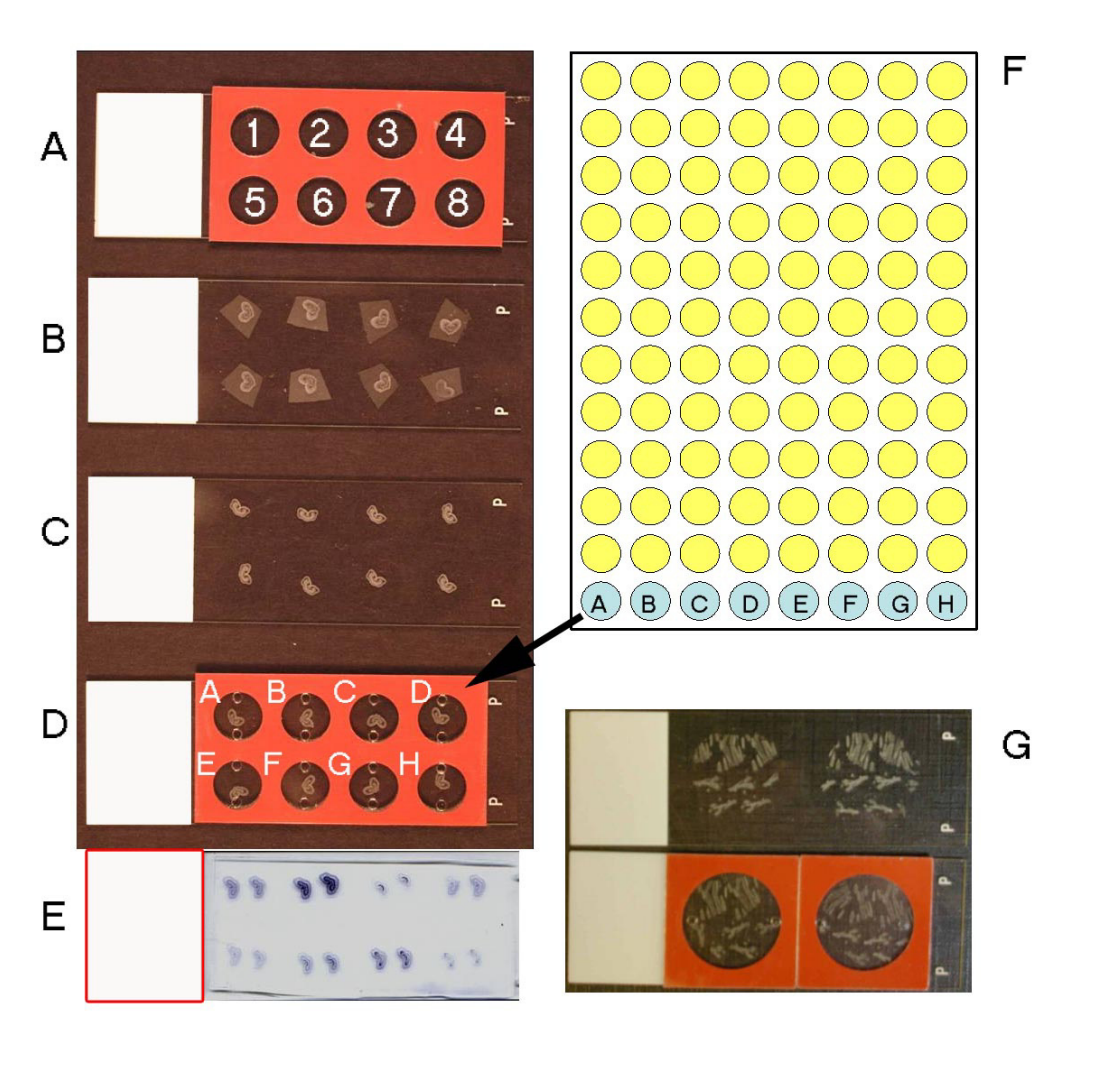
Arrangement of sections and organisation of probes: (A) Silicone isolators to position sections on slides. Positions are shown 1 to 8. (B) Sections placed in postions 1 to 8, dried and shown after removal of silicone isolator. (C) Sections after de-waxing step. (D) Sections in hybridisation chambers. A-H cross refers to plate position of the probes (F). (E) Sections after colour development. (F) Organisation of probes in 96 well plate. (G) Organisation of *Arabidopsis *sections showing larger format hybridisation chamber on lower slide.

Orientating and mounting in block for sectioning are skilled steps that normally require manual processing and these steps are therefore identical in both manual and automated protocols. Blocking up was carried out by hand to ensure favourable orientation but is facilitated by a dedicated embedding station. Sectioning cannot be automated due to the need to continuously assess section quality. However, the arrangement and number of tissue sections on the slide was made uniform using adherent, but removable, silicone isolators (Figure 2). This allowed the parallel screening of multiple probes on the same slide containing up to eight sections, each section in an isolated well.

When the sections had adhered to the slide, and the silicone isolators were removed, the slides were treated to remove wax and to make the sections receptive to the hapten-labelled probes. These down-stream treatments are universal to all tissues and appropriate for automation. However, these treatments are complex and we evaluated which ones were functional (i.e. produced an enhanced, yet specific signal) and which were redundant. Using a training set of probes and the traditional manual protocol, we systematically eliminated or reduced each step in the protocol and visually evaluated the final result. Slides were processed in parallel but with a proportion subjected to a protocol that omitted one or more steps normally used. This led to a reduction in the number of ethanol dehydration steps and elimination of the 're-fixation" step after proteinase K treatment. Some steps, although not absolutely essential, appeared to enhance the reliability of the process and these were retained: for example, acetic anhydride treatment was found to reduce background (especially on poly-lysine coated slides) and we increased the time allowed for de-waxing in xylene while applying agitation using the VP2000 slide processor (see below).

Finally, using a basic open-plan slide processor (VP2000), common in medical cytology labs, a reduced section-processing protocol with essential steps only was automated and made completely hands-off. This also eliminates much of variation possible in a multi-step, closely timed procedure and led to more reproducible signals.

#### (ii) Probe-making

**Figure 3:**
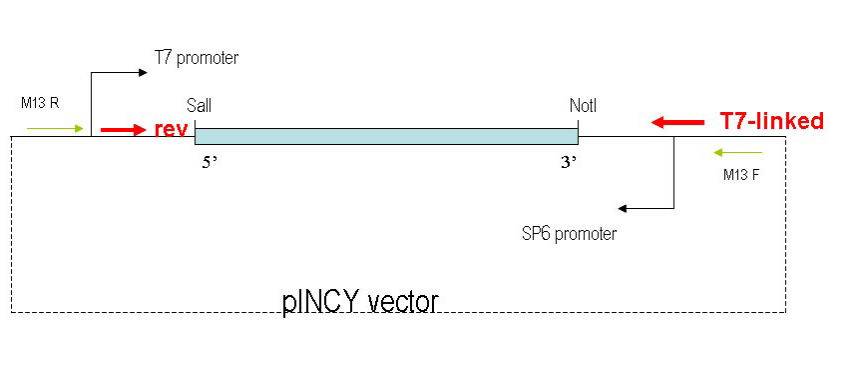
Schematic showing the production of templates for probe labelling. PCR reactions used cDNAs in pSPORT-derived vectors as template with a T7-linked 3' primer and a 5' primer based on vector sequence. T7 RNA polymerase was used to make all antisense probes.

In the manual protocol, individual probes were made from linearized plasmids. This necessitates the analysis of each clone for suitable restriction sites. To eliminate time-consuming individual analysis, we used a PCR strategy to produce linear plasmid inserts for probe transcription. This allows the production of large number of probes in parallel, particularly if the original clones are in a common vector. For a collection of genes inserted in the same orientation in a common vector, as found in most gene libraries, a common pair of primers can be designed to the flanking regions of the vector and used to prepare all probes. Thus, for the wheat cDNAs we designed a pair of primers against the flanks of the polylinker region for the pINCY vector (a derivative of the pSPORT vector from Invitrogen). The 3' primer contained a T7 RNA polymerase transcription site (see Figure 3). Thus, all the antisense probes were amplified using the same primer pair and subsequently all transcribed with T7 RNAP (T7 RNA Polymerase). This strategy was scalable: templates were produced in batches of 96 (using a liquid dispensing Q-bot) and yield was estimated on a 96 well multi-slot E-gel (Invitrogen).

We also assessed the yield of different RNA polymerases. Although *in vitro *transcription is possible with any one of T7, T3 or SP6 RNAPs, T7 was undoubtedly the most efficient providing high yields of almost every cDNA. SP6 was found to be the least consistent, but could produce reasonable yields for a low proportion of clones. We did not investigate this further and routinely used T7 RNAP.

Next, we minimised the protocol for the *in vitro *labelling of the PCR products to the following essential steps only: transcription with dig-UTP (digoxigenin uridine triphosphate) for two hours, immediate hydrolysis for 30 minutes for all probes and immediate precipitation with ammonium acetate (which also neutralises the carbonate hydrolysis buffer) and ethanol for 30–60 minutes. The labelling procedure was performed in 96-well plate format and took a total of four hours. To monitor transcription efficiency, a small aliquot from each batch of transcription products was run on an agarose gel to ensure that transcription is working and we repeated this test after the hydrolysis step.

The manual protocol describes individual hydrolysis times for each probe depending on the length of the DNA but we have found that once probes are hydrolysed to below a certain length, more detailed definition of an optimal probe size is not required. Hydrolysis fragments the probe and, in theory, the small fragments can access the RNA within tissue sections. However, subjecting the same transcript to several various hydrolysis incubations had little effect on signal strength. As template lengths ranged from 0.5 to 1.5 kb, we subjected each labelled transcript to the same hydrolysis treatment. When assessed on an agarose gel, all probes produced small fragments within a comparable range. Labelled transcription products were resuspended in TE and could be stored at -70°C where they remain usable for at least several months.

Accurate quantification of probes by dot-blot requires serial dilution but a simple qualitative test using a 1:100 dilution of each probe is usually sufficient to assess probe quality. If labelling is detected at this dilution, the same dilution in Hybridisation Solution (HS) can be denatured briefly and added to the slide for hybridisation. Since the probes are single stranded RNA, they are very prone to degradation during storage. Stability is much enhanced by diluting probes directly into the HS and storing them at -20°C: the HS contains 50% formamide which inactivates RNases.

#### (iii) Hybridisation and signal detection

At the end of the pre-treatment in the VP2000, the slides were dried and the silicone incubation chambers were carefully applied (Figure 2D). 40 μl of HS/probe mix was applied to each chamber in a standardised order from 96 well plate to slide (i.e., plate A1-A8 to slide 1 etc.,) such that 96 probes (including controls) can be applied to 12 slides (Figure 2F). Hybridisation was overnight at a standard 50°C in a conventional incubator on horizontal slide racks.

Use of hybridisation chambers with the same dimensions as the isolator allowed at least 8 different probes to be applied to a single slide. A range of sizes of isolator and hybridisation chambers are available and different sizes may be better suited to different tissue types (Figure 2G). Use of glass coverslips for incubation steps in the manual protocol normally requires prolonged, but gentle, washing to remove coverslips without damaging the underlying sections. However, the chambers can be quickly removed and the slides were loaded immediately for a short washing step in the VP2000. In addition, we also evaluated the various post-hybridisation steps and found that the RNase treatment step could be omitted completely. The RNAse step is thought to decrease background staining in ISH but slides prepared with or without RNase are essentially identical.

After standard incubations in blocking solution and Anti-DIG-AP (alkaline phosphatase) antibody, the slides were developed colorimetrically and a sample of the output is seen in Figure 2E. Using the colorimetric system rather than fluorescence means the progress of development can be monitored under a dissecting microscope and the problem of plant tissue autofluorescence is avoided. All reactions were stopped simultaneously, and all slides mounted permanently and could be stored for image-capture on the microscope.

#### (iv) Processing of results

Previous methods using one probe per slide meant that microscopic analysis was time-consuming and involved positioning many individual sections for optimal picture quality. In the automated protocol, with uniformly arranged sections representing multiple probes on every slide, image capture can be streamlined and has the potential for further automation.

#### (v) Data collection and storage

**Figure 4:**
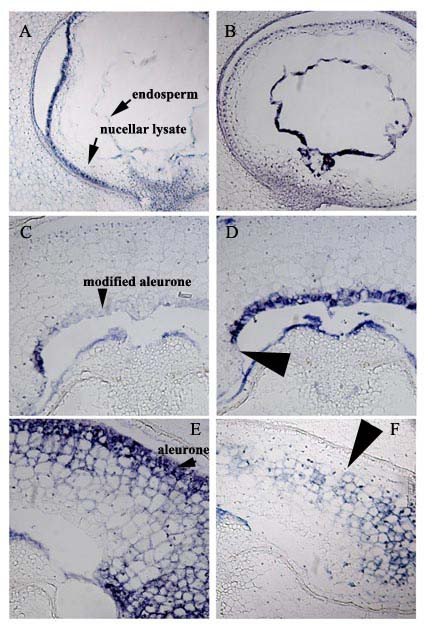
Examples of gene expression patterns in developing wheat grains. ID numbers indicate the Incyte gene code and can be used to search the wheat *in situ *database at SCRI  (user: guest; password: wheatinsitu). (A) transcript detection specifically in the nucellar epidermis at 3DAA (ID 702007486). Arrowheads indicate no expression in the innermost nucellar lysate or the coenocytic endosperm. (B) coenocytic endosperm at 3DAA (ID 702038349) (C) peripheral cells of the modified aleurone only show transcript accumulation of an unknown gene at 9DAA (ID 701965703) (D) gene expression of a plantacyanin orthologue throughout the modified aleurone at 9DAA (ID 702044644). This section is from the same grain as in (C) and the arrowhead indicates that the most peripheral cells showing signal in (C) are not expressing plantacyanin. (E) a proteinase inhibitor is expressed strongly in the outer layers of the central endosperm at 9DAA (ID 701965839) (F) in contrast to (E), a gliadin storage protein gene is expressed throughout the central endosperm but not in the outermost layers (indicated with arrowhead; ID 702007003)

Images are collected in order and were directly linked to spreadsheet or database records of the probes used in the screening. In a recently published experiment on wheat seed development [19], batches of 96 probes were used to generate 288 images of 3 developmental stages. These images were labelled in order A1-96, B1-96 and C1-96 for each plate and stored accordingly. Representative results from the wheat project are shown in Figure 4A–F and are taken from different stages screened to show signal detection in the varied cell layers in the endosperm and surrounding tissues. These results illustrate that the automated protocol provides at least cellular resolution and in many cases, subcellular. Very specific patterns are defined within even thick-walled cells (such as the transfer cells of the nucellar projection and modified aleurone), which are likely to be recalcitrant to whole-mount procedures. Various other cell types are equally well stained with appropriate probes: the small cuboid cells of the young integument layers, the highly-vacuolate cells of the 9 DAA (days after anthesis) endosperm and the early multi-nucleate, but unicellular, coenocyte.

**Figure 5:**
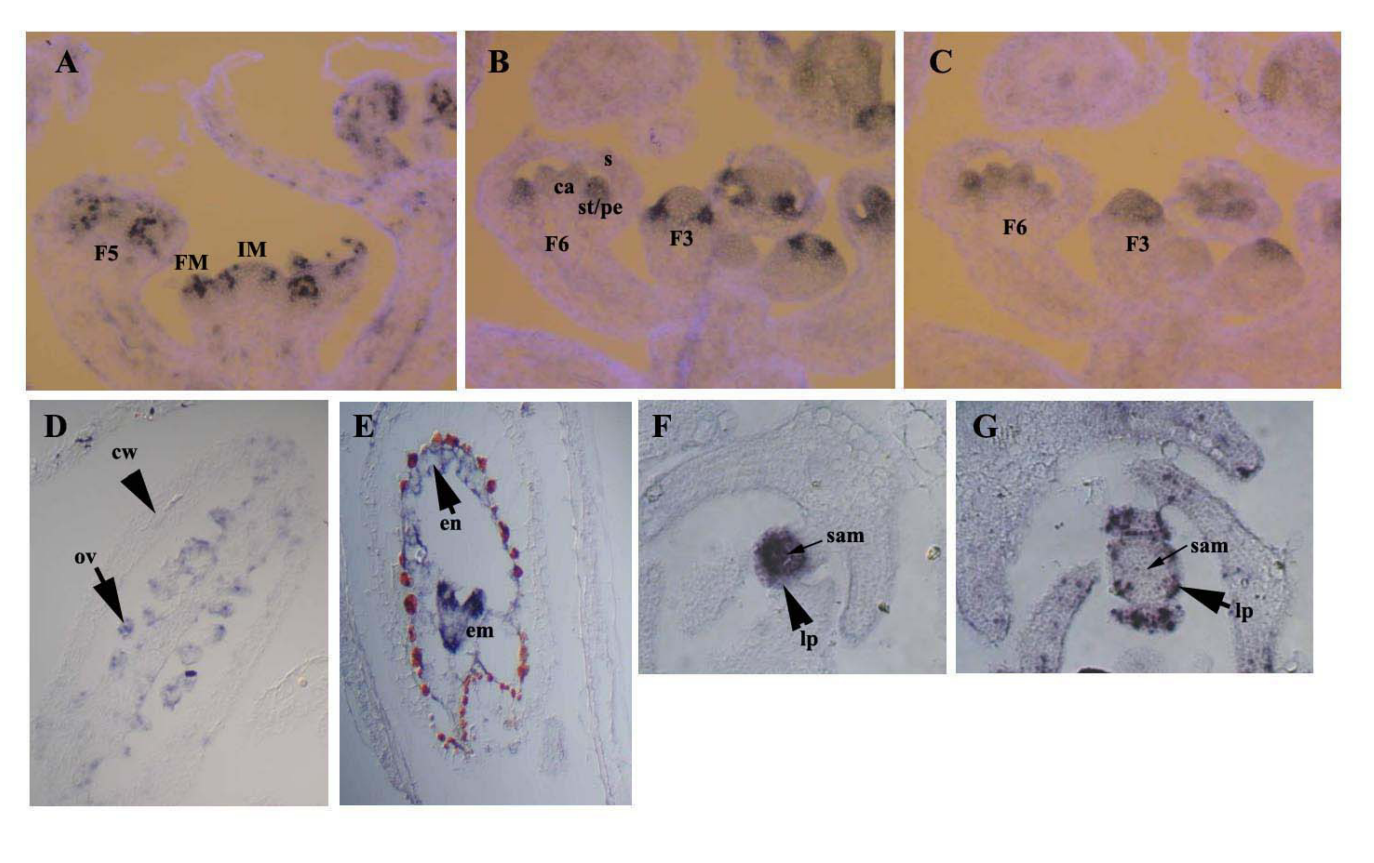
The automated ISH protocol on *Arabidopsis *tissues. Developing flowers (A-C), developing siliques (D), ovules (E) and transverse sections of the shoot apical meristem (SAM) of 10-day seedlings (F-G). Probes used are for *histone H4 *(A, D, E, G), *AP3 *(B), *AG *(C) and *stm *(F). (A-C) were counterstained with the cell wall dye, Calcofluor, which produces a light blue colour. Expression of *AP3 *and *AG *in serial sections (B and C) shows the distinct patterns of expression in the petal/stamen primordia for the class B *AP3 *gene and in the carpel primordia for the class C *AG*. In (D) arrows indicate expression of *histone H4 *in the developing ovules but by this stage there is no expression in the silique/carpel wall. Expression is also detectable in the endosperm of the developing ovule as well as in the cotyledons and root meristem of the embryo (E). In (F) an arrow indicates absence of *stm *expression in the leaf promordia but *histone *is expressed here and in the slightly older leaves (G). IM, inflorescence meristem; FM, floral meristem, number indicates the approximate flower stage; s, sepal; ca, carpel; st, stamen; pe, petal; cw, carpel wall; ov, ovule; en, endosperm; em, embryo; sam, shoot apical meristem; lp, leaf primordium.

**Figure 6:**
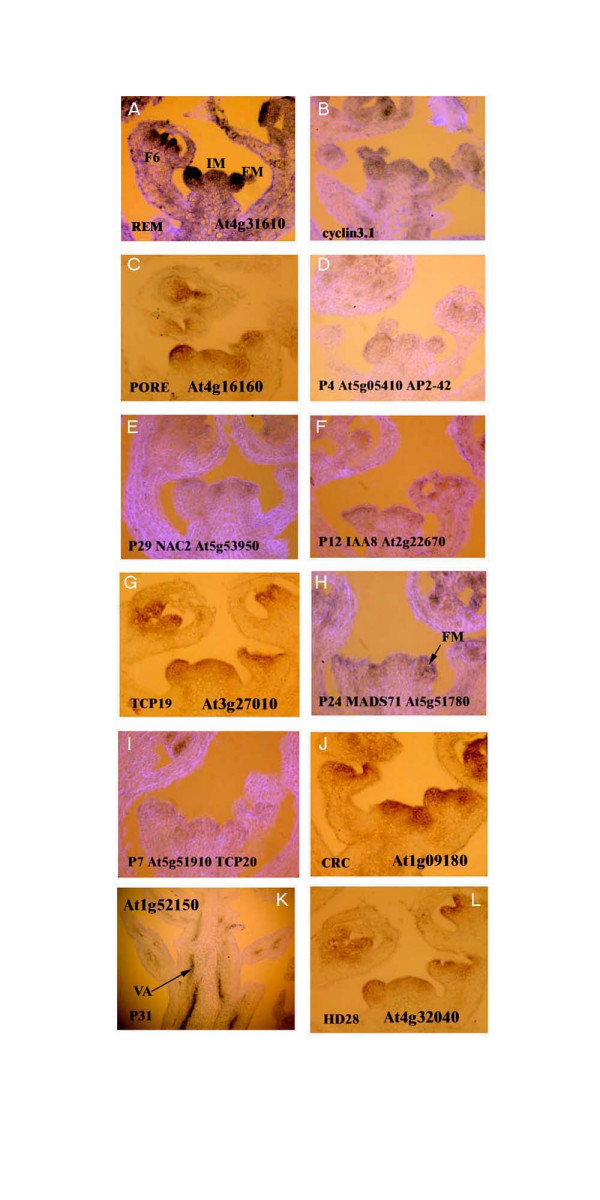
A selection of gene expression patterns (mostly transcription factors) in young *Arabidopsis *flowers (A-L). AGI gene annotations accompany each figure in the panel. Expression in young floral meristems is strong in A, B, C, G, J and L. The arrow in H indicates weaker but specific expression in the floral meristem and in K there is strong expression of *CNA *in the vasculature (VA). A, B D, E, F, H, and I have been counterstained with the cell wall stain, Calcofluor, which produces a light blue colour.

We also evaluated the protocol on other species, including *Arabidopsis*. Floral meristems were fixed and processed using the automated procedure and probes prepared in 96-well format. Using a training set of 4 previously characterised genes *histone H4*, *AP3*, *AG *and *stm *[18,20-22] (Figure 5) alongside genes encoding a variety of other cellular functions, we show that this approach has the potential for systematic spatial analysis of gene expression in a model organism, with at least cellular resolution (Figure 6). The expression patterns of a set of ten genes are shown in figure 6 includes some previously characterised genes including *AtREM1*, encoding a plant-specific regulatory protein and expressed in the floral meristems [23] (Figure 6A), *CRABS CLAW*, encoding a helix-loop-helix regulator of carpel development [24] (Figure 6J) and the recently described *CORONA *gene encoding a leucine zipper regulator of vascular tissue [25] (Figure 6K). These patterns are very similar to previously published results, indicating that the new protocol is robust and can be used for a range of genes without specific tailoring to each gene.

## Conclusion

We describe a semi-automated system for highly parallel processing of ISH. We have introduced a substantial degree of automation to produce a system for performing high-throughput RNA-ISH on hundreds of plant tissue sections simultaneously without loss of resolution, specificity or sensitivity. This slide processing system has a capacity of at least 96 probes per week/per person for multiple developmental stages or experimental treatments and, with the exception of imaging, is scalable. Therefore, it is now feasible to contemplate genome-wide spatial analyses of gene expression at cellular resolution in both crop and model plant species.

To achieve genome-wide coverage in any species, certain prerequisites are necessary. First, sequences representing the expressed genes must be suitable for making probes and be available in an organized format. Second, the remaining manual steps must be further streamlined and, ideally, automated. Finally, automated image collection, analysis and quantification methods need to be developed.

Several projects are currently underway whose collective aim is to provide the expressed *Arabidopsis *genome as organized libraries of clones. Thus, a large proportion of *Arabidopsis *genes are currently available as trimmed ORFs (open reading frames) from SALK  and ESTs (expressed sequence tags) from ABRC (Arabidopsis Biological Resource Centre) , or as 3' UTRs (untranslated regions) or as groups of structurally related genes from special projects such as REGIA (**Re**gulatory **G**ene **I**nitiative in **A**rabidopsis) . These collections tend to present genes in a consistent format that is amenable to automation. In most crop species, where the genomes are as yet incomplete, large EST collections are available. However, even these can provide useful information when used in conjunction with microarrays, where gene expression data can be confirmed and resolved to individual cell types and tissue layers [19].

Throughput is somewhat lower in *Arabidopsis *than in wheat, largely due to the time required for sectioning the smaller and more heterogeneous tissues. The proportion of usable sections is less and the tissue complexity requires greater imaging times. However, the smaller size of *Arabidopsis *facilitates whole mount approaches [3,11] and, used with confocal microscopy and fluorescent imaging, this could completely eliminate manual sectioning without compromising either resolution or throughput.

Further automation is therefore required. Recent developments in slide processing have provided improved and more cost-effective slide processors that automate all the steps from de-waxing to mounting, including hybridisation (unpublished results) but imaging and analysis remain significant rate-limiting factors. Automated imaging and analysis involving machine-learning, are essential to extend this approach to the analysis of whole genomes. Such approaches have been developed for the analysis of tissue microarrays in the analysis of protein expression in various cancers, though these technologies still involve protein immunohistochemisty more than mRNA *in situ *hybridization [26-28]. However, as they employ a similar colour-based detection system, the technology should be transferable.

## Methods

### Preparation of plant material

Wheat plants (variety Savannah) were grown under controlled environment conditions (16°C, 16 h light) and ears tagged daily at anthesis. *Arabidopsis *Col-0 was grown in glasshouse under a 16-hour light regime. Wheat grains harvested at 3, 6 and 9 DAA were trimmed and *Arabidopsis *floral meristems were removed just after bolting. All tissues were fixed in 4% paraformaldehyde, then transferred to the Tissue Tek VIP (Vacuum Infiltration Processor, Sakura) for an automated fixation/dehydration/infiltration process as follows: fixative 6 h 35°C; 70% ethanol 1 h 35°C, 80% ethanol 1.5 h 35°C; 90% ethanol 2 h 35°C; 100% ethanol 1 h 35°C; 100% ethanol 1.5 h 35°C (repeat for 2 h); xylene 0.5 h 35°C (repeat for 1 h and again for 1.5 h); wax 1 h 60°C (repeat once, then again twice for 2 h). All steps were performed under vacuum. Samples were then transferred to the Tissue Tek Embedding Console for embedding in paraffin wax.

### Section preparation

Sections (14 μm) from wheat samples at the required stages and 8 μm sections from floral meristems were cut on a Leica Microtome (RM2125RT) and ordered on polysine slides containing the silicone isolators (Grace Biolabs). After drying down at 42°C overnight suitable sections were selected for pretreatment.

Pretreatment steps were performed using the VP2000 Slide Processor (Vysis) using the following program: xylene 20 min (twice); 100% ethanol 10 min, then through a 95%, 85%, 50%, 30% ethanol series (2 min each), PBS (3 mM NaH_2_PO_4_, 7 mM Na_2_HPO_4_, 130 mM NaCl) 3–4 min; proteinase K (2–3 μg/ml in 100 mM Tris, 10 mM EDTA pH7.5) 30 min at 37°C; glycine (0.2%) 2 min; PBS 3–4 min; acetic anhydride (0.5% in 0.1 M triethanolamine pH 8) 10 min; PBS 3–4 min, then back through the ethanol series. Slides were completely dry at this stage and could be stored at 4°C until hybridisation.

### Generating templates and labeling probes

Wheat cDNAs for screening are supplied as inserts in a vector derived from pSPORT1. Primers were designed in order to append a T7 RNAP site to the 3' end of the insert with the other primer nested inside the native vector T7 RNAP site; T7.2 5' GAATTGTAATACGACTCACTATAGGGCCAGTGAATTGAATTTAGG 3' and R7.2 5'AGGGAAAGCTGGTACGCCTGC 3' (T7 RNAP promoter binding site underlined). *Arabidopsis *histone H4 was amplified from pBluescript and AP3, AG, REM from pGEM vectors with universal forward and reverse primers for subsequent transcription with T7 RNAP. PCR reactions were performed with the following cycle: 94°C 3 min, then 30 cycles of 94°C 45 s, 63°C 45 s and 72°C 1.5 min, final extension of 72°C for 6 min. For 96-well plates PCR-product purification was done using the Montage Clean-up Kit (Millipore).

*In vitro *transcription was performed in 10 μl reactions for 2 h at 37°C in the presence of digoxigenin-UTP (Dig-UTP)-nucleotides (0.35 mM). Hydrolysis was carried out immediately in 100 mM carbonate buffer pH10.2 at 60°C for 30 min, and products precipitated in 2.5 M ammonium acetate and 3 vol absolute ethanol for 1 h at 4°C. Plates were centrifuged at 4000 rpm for 30 min and pellets resuspended in 30 μl TE (100 mM Tris, 10 mM EDTA) buffer. Dilutions (100 x) were made in water and 1 μl of each spotted on nitrocellulose for dot-blot: 30 min in blocking solution (Sigma), 30 min in anti-DIG-alkaline phosphatase (Roche); 5 min wash in TBS (10 mM Tris, 250 mM NaCl); 5 min in AP-buffer (100 mM Tris, 100 mM NaCl pH 9.5; 50 mM MgCl_2_) and developed as described above until signal was sufficient. All probes were then diluted 100-fold in hybridisation solution (300 mM NaCl, 10 mM Tris pH 6.8, 10 mM NaPO_4_, 5 mM EDTA, 50% formamide, 5% dextran sulphate, 0.5 mg/ml tRNA, 1 × Denhardt's, 0.1 mg/ml salmon testis DNA) and maintained stably at -20°C until hybridization.

### Hybridisation and washing

Chambers (Grace Biolabs) were applied securely to the slides (after pre-treatment) and probes (diluted in hybridisation solution) were applied to one well (2 sections) for the 3 stages individually. Coverslips were placed on the chambers to prevent evaporation and hybridisation was performed overnight in a 50°C incubator.

Chambers were removed and slides arranged in the VP2000 for washing program: 15 min in 2 × SSC (0.3 M NaCl, 0.03 M Na citrate), 50% (v/v) formamide at 40°C; 40 m in the same at 50°C; 20 min in 1 × SSC, 50% (v/v) formamide at 50°C (all steps with constant agitation); 5 min in 1 × SSC at room temperature; 5 min in 1 × TBS at room temperature. Then slides were transferred into trays for staining: 1% blocking solution (Roche) in TBS 1 h, 1 × TBS containing 1/3000 dilution of Anti-DIG AP and 0.05% (v/v) Tween-20 1 h; 4 × 10 min washes in 1 × TBS; 5 min in AP-Buffer (0.1 M Tris, 0.1 M NaCl, 50 mM MgCl_2_); developed in AP-Buffer containing NBT (0.1 mg/ml) and BCIP (0.075 mg/ml) for a maximum of 24 h. Slides were then washed several times in water to stop the reaction followed by sequential washes in 70% and 100% ethanol to remove excess stain (the duration of the ethanol washes depends on the level of colour development which was monitored by eye). Slides were then allowed to dry and permanently mounted in Entellan (Merck).

### Image capture and analysis

One section for each stage for each probe screened was photographed on a Nikon E800 microscope using a digital camera under brightfield conditions for wheat sections and with UV filter for the calcofluor-counterstained *Arabidopsis *sections. Images were recorded sequentially as ordered on the slides. Magnifications and camera settings remained unchanged for all images through all stages for wheat section and likewise for floral meristems.

## Competing interests

The author(s) declare that they have no competing interests.

## Authors' contributions

SD developed the system described, performed all work for the wheat experiments and wrote the manuscript; JC and BC provided technical assistance on adapting the technique to *Arabidopsis*; JHD, PS and LD were co-supervisors; JHD co-wrote the manuscript.

## Supplementary Material

Additional File 1 Detailed high-throughput *in situ *hybridisation protocol. An illustrated text document is attached with a protocol written in step by step detail for people working at the bench. Click here for file
