# Supplementary material for: A streamlined method for systematic, high resolution in situ analysis of mRNA distribution in plants
Source: Plant Methods. 2005 Oct 6;1:8. doi: 10.1186/1746-4811-1-8 (PMC1280931; doi:10.1186/1746-4811-1-8)
Supplement: Additional File 1 — Detailed high-throughput in situ hybridisation protocol. An illustrated text document is attached with a protocol written in step by step detail for people working at the bench. [file 1746-4811-1-8-S1.doc]

***In situ* protocol**

**I**

## Plants

- tagging

- harvesting

**II**

## Probe template

- PCR with T7-primer

- purify products

# Tissue preparation

**-** Fixation

- Wax embedding

**Pre-treatment**

- De-waxing

- protease treatment

- acetylation

## Probe labelling

- *in vitro* transcription with Dig-UTP

- Hydrolysis

Probe preparation for hybridization

- quantification

- heat denaturation

### Hybridization

- solution containing formamide, salts, blocking agents

#### Immunological detection

- staining with Anti-Dig

- colorimetric substrate

- slide mounting

**Slide preparation**

- sectioning

- drying

**III**

## Stringency washes

- formamide, temperature, salt

### Microscopy

- Light microscopy
- Brightfield/Darkfield
- DIC (Nomarski)

IV

## Data Handling

- recording results (images and comments)

- database design

**V**

**I. Tissue Preparation**

**I.1 Tissue Fixation**

Fixative:

4% (w/v) paraformaldehyde

Prepare in fume hood

For 2.5 l fixative, add 100 g paraformaldehyde to 1.25 l water and heat to 50-60°C for 30 min. Add drops of 1M NaOH until completely dissolved. When solution has cooled to about 30°C add 1.5 l of 2 x PBS (phosphate buffered saline).

Tissue should be harvested and trimmed just before placing in fixative.


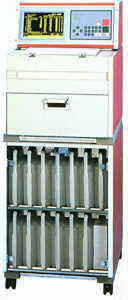
Place tissues in cages and place all in the Tissue-Tek Vacuum Infiltration Processor. Transfer the 2.5 l of fix solution to the first container of the Processor and initiate the following fix cycle:

Fixative 6 h 35°C

70% Ethanol 1 h 35°C

80% Ethanol 1.5 h 35°C

90% Ethanol 2 h 35°C

100% Ethanol 1 h 35°C

100% Ethanol 1.5 h 35°C

100% Ethanol 2 h 35°C

Xylene 0.5 h 35°C

Xylene 1 h 35°C

Xylene 1.5 h 35°C

Wax 1 h 60°C

Wax 1 h 60°C

Wax 2 h 60°C

Wax 2 h 60°C

This machine operates on an enclosed system with internal storage bottles containing all the relevant solutions and exchange of solutions for the cycle performed automatically. The volume of solution in each bottle is 2.2 l. The fix solution is changed each time a run is performed whereas all other solutions are used several times and only changed when required. When the solutions need to be changed is indicated on the machine.

**
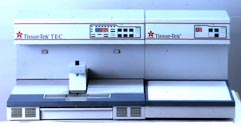
**

**I.2 Tissue Wax**

**Embedding**

Tissue is removed from VIP and transferred to the wax basin of the Tissue Tek Embedding Station. Into the warm mould, pour a thin layer of wax; orientate tissue quickly and then as this layer starts to set, add more wax until the mould is full. Apply plastic backing and allow to set on adjacent cold-plate. Store in the fridge or cold room.

**I.3 Tissue Sectioning**

## Slide Layout

**
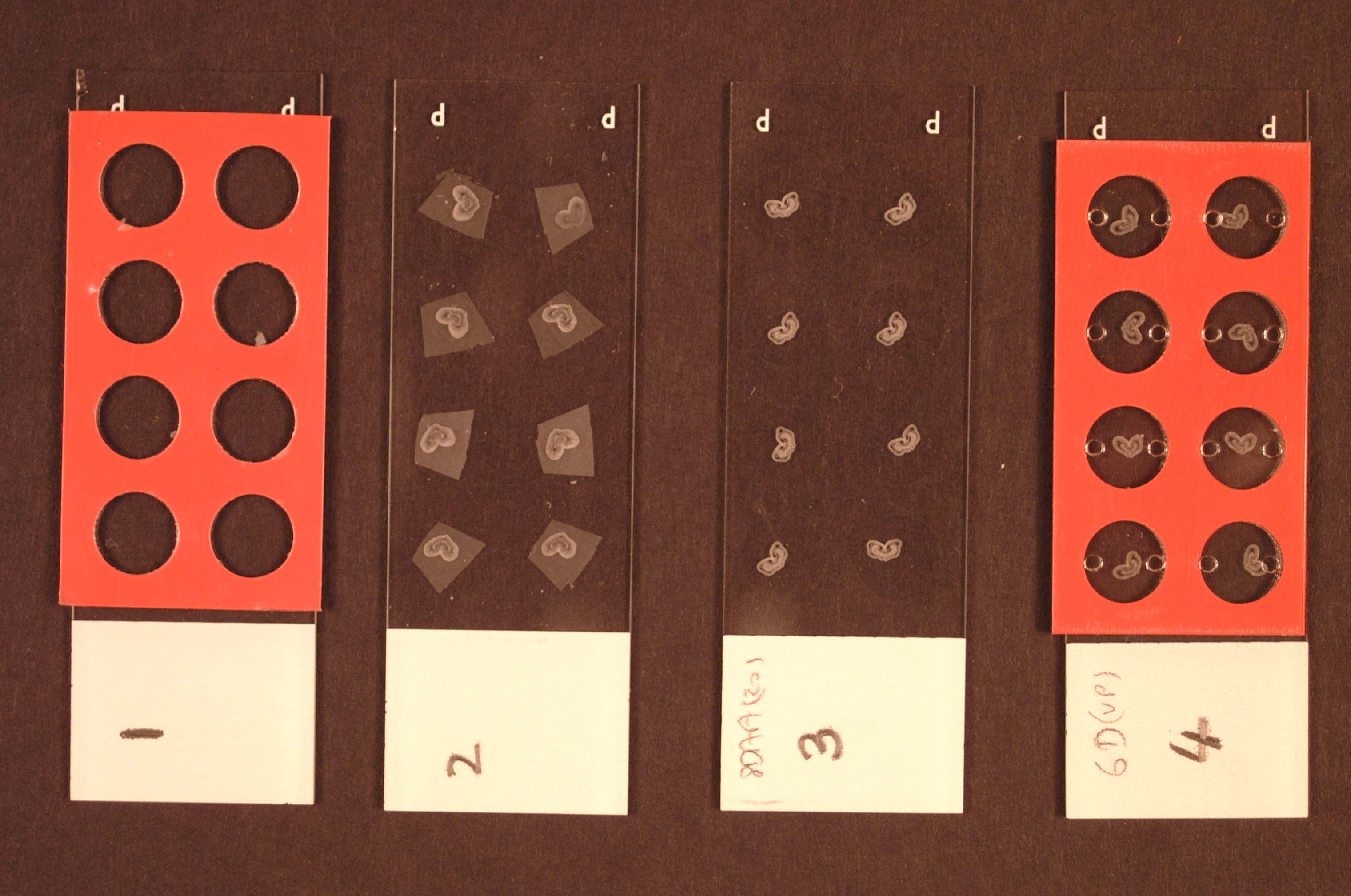
**

Section/probe order isolator

# a1

**7/1**

**1**

**1**

Slide ID

**Date**

**Slide order**

**
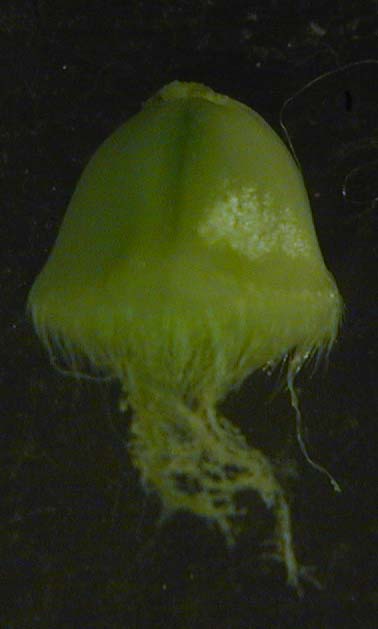
**

**Top (embryo)**

Slide format (see above):

**stop**

- maximum of 3 stages are proposed for each

screening run; a, b and c

**start**

- probes designated 1-8 will be on slides a1, b1

and c1; probes 8-16 on a2, b2 and c2 etc.

Sectioning:

-

-14 μm thick

**bottom**

- 2 per well/16 per slide

- grains trimmed at “bottom end” (allows

wax penetration and flat surface for embedding)

- Sectioned right through endosperm and sections ordered on slides.

- Check for preservation of tissue and expected morphology for that stage.

Sectioning is best performed in a cool environment, about 18-22^o^C.

Label slides with pencil on frosted end.

Cut the wax block to a small trapezoid shape keeping in mind the dimensions of the wells and that we aim to fit two sections in each.

Mount the block so that the longer side is to the bottom and therefore hits the blade first.

Cut ribbons of sections at about 14 μM thickness (optimal thickness for easy handling of many sections).

Use a fine artists brush to move ribbons to clean paper toweling.

Cut the ribbons every two sections with a clean razor blade and place the “double” sections in each “well “.

Float ribbons on to water on pre-coated polysine slides (BDH) and place in oven to dry down for at least 24 h.

# I.4 Pre-treatment of sections in the VP2000

Timings may seem generous but as the programme is automated and captive time is nil. All solutions derived from autoclaved stocks diluted with sterile water.

Slides are loaded in the rack


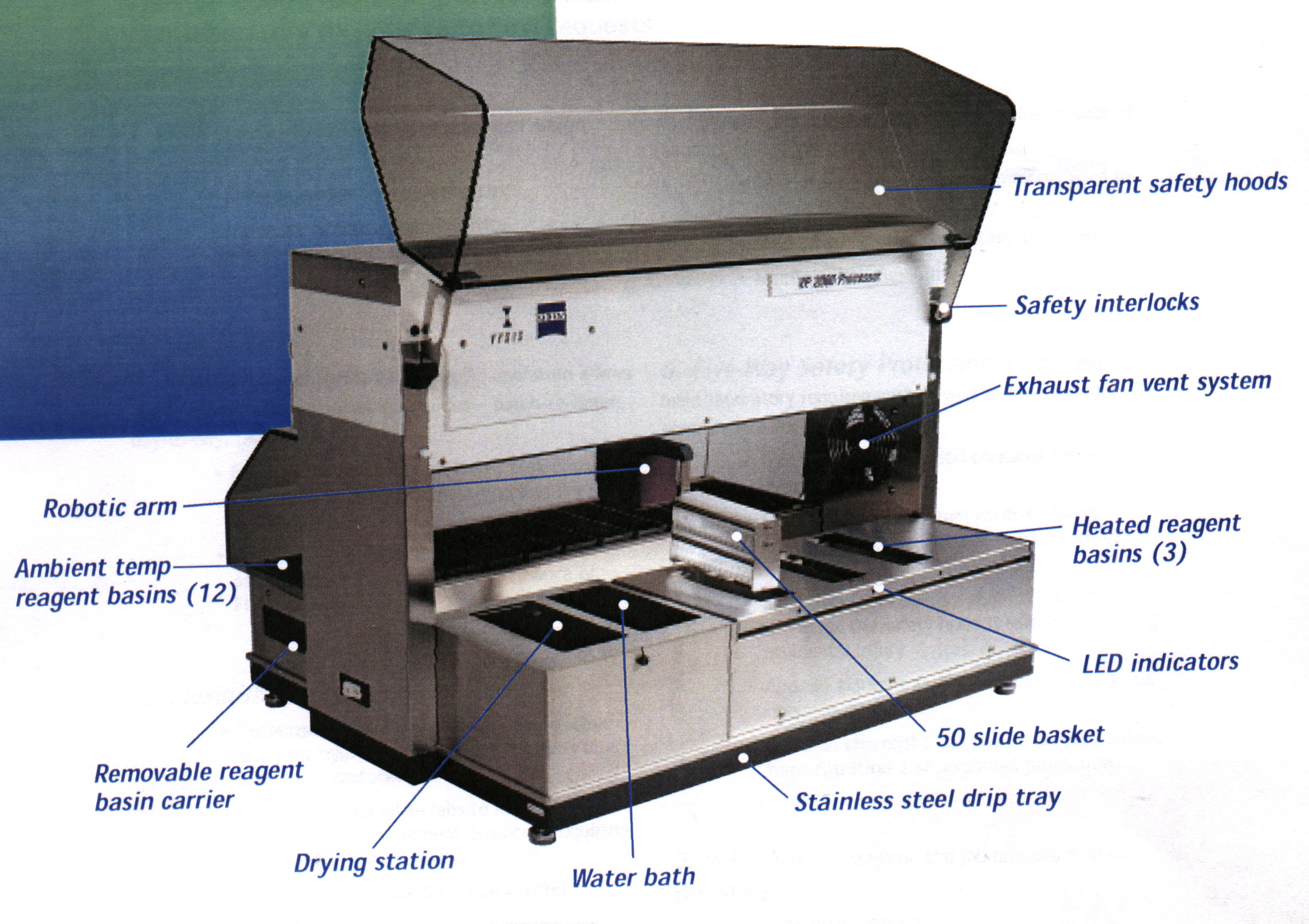
(maximum capacity of 50) and

fixed to machine.

The following protocol is loaded:

1. Xylene 1 20 min

2. Xylene 2 “

3. Xylene 2 3 min with agitation

4. 100% ethanol 10 min

5. 100% ethanol 1 min with agitation

6. 95% ethanol 2 min

7. 85% ethanol “

8. 50% ethanol “

9. 30% ethanol “

10. PBS-1 3 min

11. PBS-2 1min with agitation 18. PBS-1 1min with agitation

12. ProtK 30 min at 37°C 19. 30% ethanol 2 min

13. Glycine 2 min 20. 50% ethanol “

14. PBS-3 2 min 21. 85% ethanol “

15. PBS-3 1 min with agitation 22. 95% ethanol “

16. TEA/AA 10 min with agitation 23. Drying Station at 25°C for 4 min

17. PBS-2 2 min

At this stage the tissue is ready for hybridisation but slides can be stored at 4°C for at least several days.

**II. Probe Preparation**

**II.1 DNA preparation** (PCR)

DNA provided as pINCY clones with SP6 RNAP promoter at 3’ and T7 RNAP promoter at the 5’ end.

**F SP6 3’ cDNA insert 5’**

PCR2 **T7** **R**

**t7.2 r7.2**

This would involve the use of the SP6 promoter to generate antisense transcripts. This has proved not to be efficient however. Therefore we generate new templates with a primer (t7.2) at the 3’ incorporating another T7 promoter.

Grow bacterial cultures containing plasmids overnight at 37°C in 96-well plates (1ml per well approx.). Transfer 10μl of culture to a fresh 96-well plate and add 90μl of water. Boil for 3 min to lyse the cells. Use 1-6μl in 100μl PCR reactions with the following cycle: 94°C 3 min, then 30 cycles of 94°C 45 s, 63°C 45s and 72°C 1.5 min, final extension at 72°C for 6 min. Check 2 μl of the products to ascertain if the PCR has worked. Where amplification is from bacterial cultures we have found it necessary to clean products before in vitro transcription. For 96-well plates this is done using the Montage Clean-up Kit (Millipore). 2 μl of all products are checked on agarose gels. However, if the starting template for PCR is plasmid, we have found that if reagents such as primers and Taq etc. are kept to minimal amounts, transcription can be performed on unpurified PCR products.

**II.2 DIG-labeling of probes by *in vitro* transcription**

10μl reactions are set-up from a Master-mix containing NTPs, RNAP etc. and the DNA is added last. Individual reactions are as follows (amounts in μl):

sterile water 2.5

5 x transcription buffer 2.0

100 mM DTT 1.0

10 x NTP labeling mix 1.0*

RNasin 0.5

T7 RNA Polymerase 1.0

*10 x NTP= 1 μl each of ATP, CTP, GTP, 0.65ul UTP (100 mM stocks), 3.5 μ l of DIG-UTP (10mM), 2.85 μl sterile water for a 10 μl stock

Aliquots of 8μl are made into 96-well plate

2 μl of PCR products added using multichannel pipette

Plates are sealed

Incubate at 37^o^C for 2 h


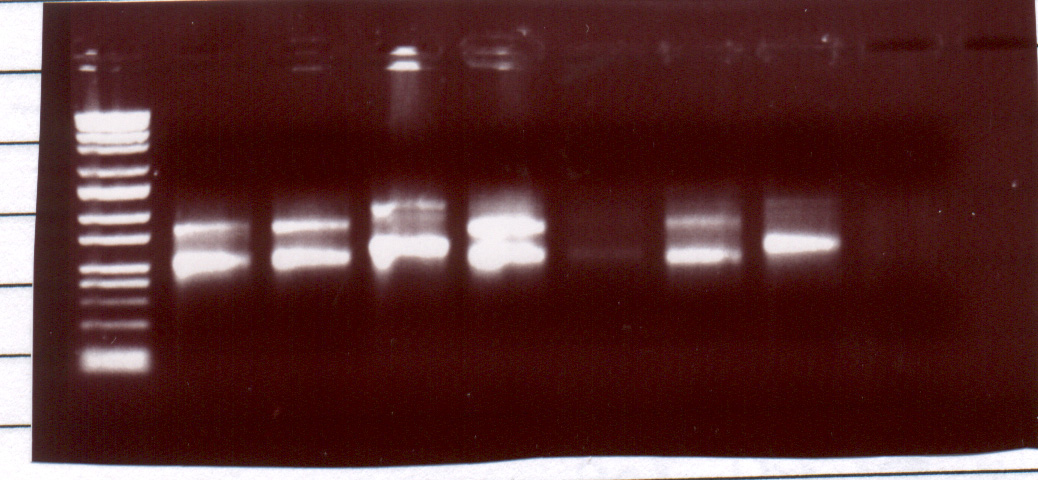
(Check 0.5 μl of several samples on agarose gel after 1 h to ascertain successful transcription)

Add 10 μl of carbonate buffer (200mM, pH10) and incubate at 60° for 30 min.

Add 10 μl of 7.5M ammonium acetate and mix well.

Add 90 μl of cold ethanol and mix. Precipitate at 4°C for 1 h.

Spin plates at 3500-4000rpm for 35 min.

Invert plates and spin briefly at 500 rpm to remove all traces of ethanol. Allow to air-dry for several min.

Resuspend pellet in 30 μl TE.

Make 1/100 dilitions of probes in water in 96-well plate. Using multichannel pipette spot 1  μl of each on Hybond membrane. Also spot dilutions of control Dig-RNA (Roche) and some experimental probes already used in *in situ*s.

Allow to dry and fix for 2 min on UV transilluminator.

Soak briefly in 1 x TBS (Tris buffered saline).

Add 1 x blocking solution (Sigma) for 30 min

Remove block and add 1/5000 anti-DIG-AP in 1xTBS for 30 min.

Wash for 5 min in 1 x TBS and 5 min in AP-buffer.

Develop for several min in NBT/BCIP (2μl/1.5 μl per ml of AP buffer).

It is difficult to accurately quantify probes by dot-blot unless one uses several dilutions of each probes and spots them individually. Therefore, dot-blotting is treated as qualitative assay for labeling only.

A

**H**


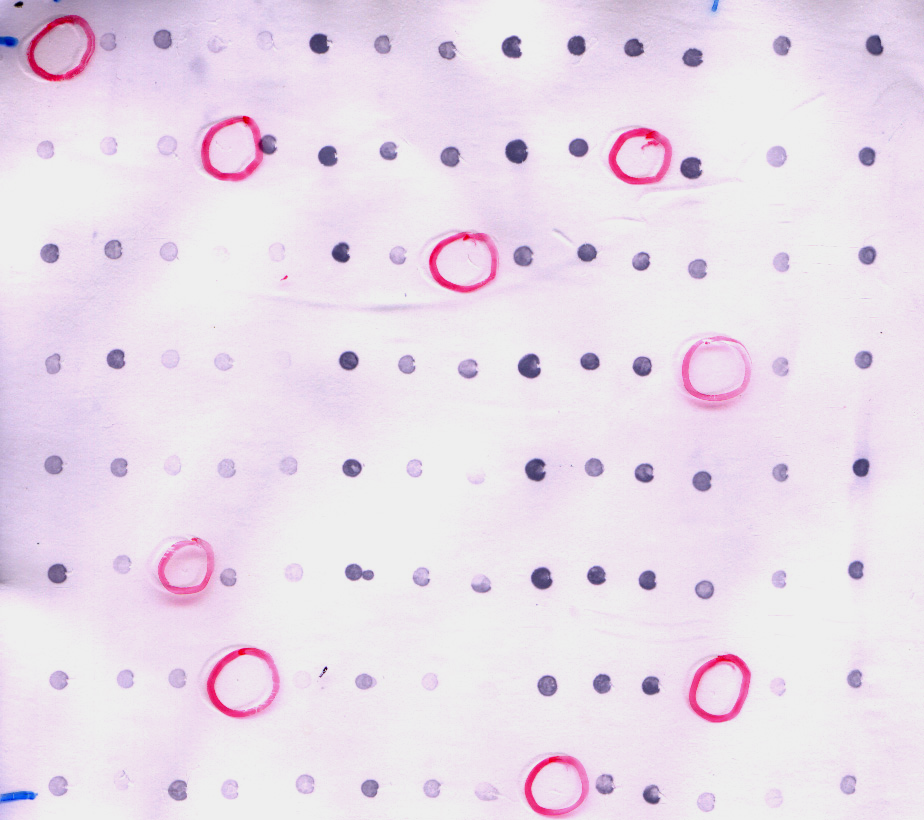


1 12 controls

(circles indicate where there are no probes present)

Dilute the probes 1/100 directly in hybridization solution (1 x Salts, 50% formamide, 5% dextran sulphate, 0.5 mg/ml tRNA, 1 x Denhardt’s, 0.1 mg/ml Salmon testis DNA) and store at -20°C.

The hybridization solution (HS) has been made in bulk and pre-aliquoted into plates so probe can be added, mixed, denatured and apply to the slides. 140 μl is pre-aliquoted (40 μl for each well and 3 wells for the 3 stages) and 1.4 μl of probe is added to each well with multichannel pipette. Mix well.

**III. Hybridization, Washing and Staining**


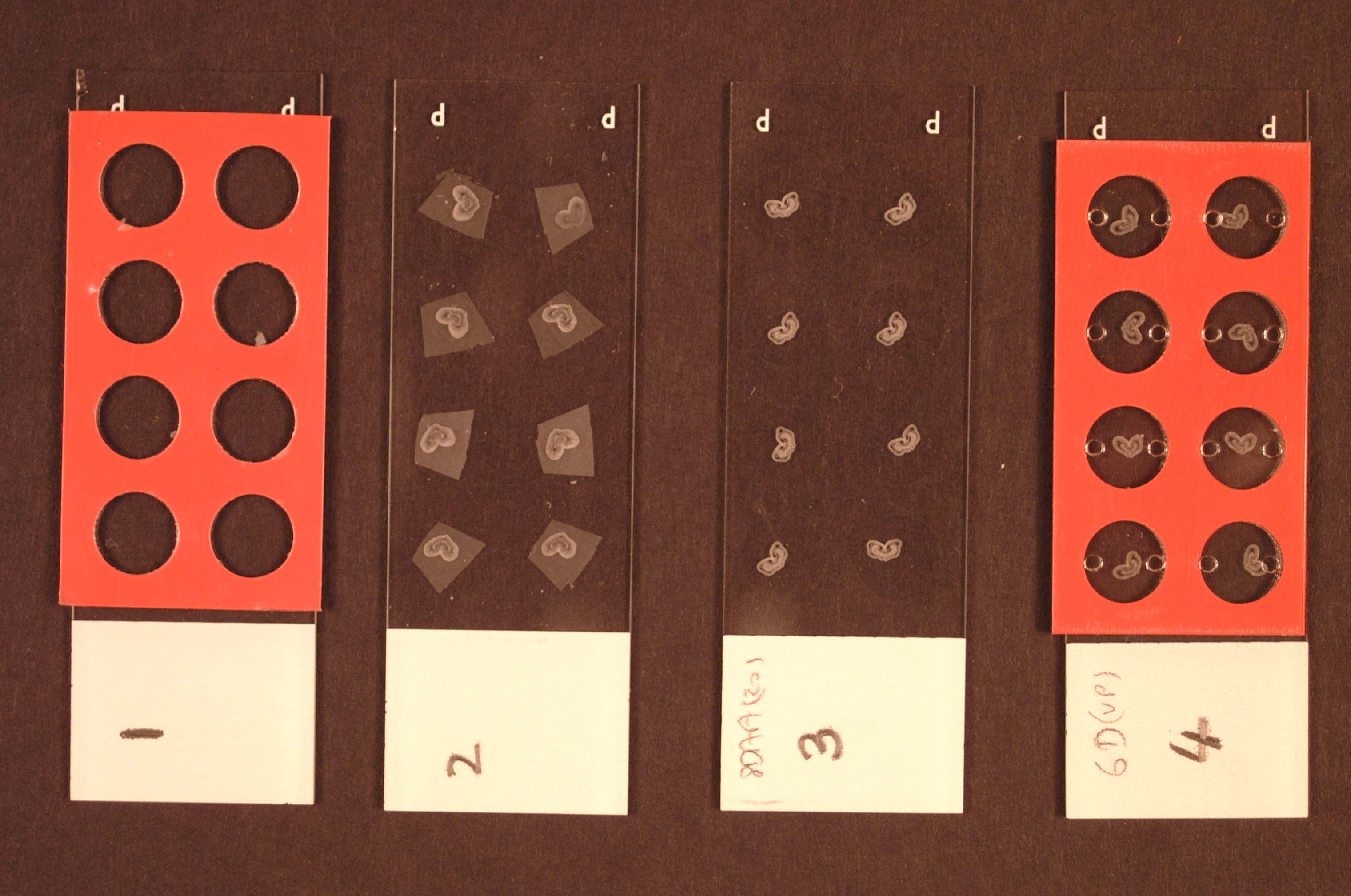
**III.1 Hybridization**

Apply the chambers carefully to the slides.

Make sure that a tight seal is formed.

Align the slides on the metal trays.

Remove the plates containing probes diluted in HS from the

-20°C freezer

Heat to 85^o^C for 2 min, cool on ice immediately and

apply 40 μl to each chamber.

Overlay with coverslip to prevent evapouration.

Hybridise overnight at 50^o^C in the oven

**Set-up:**

36 slides for 96 probes

Slide stage1 stage2 stage3

**A B C D**

**E F G H**

**A B C D**

**E F G H**

**A B C D**

**E F G H**

**A B C D**

**E F G H**

**A B C D**

**E F G H**

**A B C D**

**E F G H**

**A B C D**

**E F G H**

**A B C D**

**E F G H**

**A B C D**

**E F G H**

**A B C D**

**E F G H**

**A B C D**

**E F G H**

**A B C D**

**E F G H**

**A B C D**

**E F G H**

**A B C D**

**E F G H**

**A B C D**

**E F G H**

**A B C D**

**E F G H**

**A B C D**

**E F G H**

**A B C D**

**E F G H**

**A B C D**

**E F G H**

**A B C D**

**E F G H**

**A B C D**

**E F G H**

**A B C D**

**E F G H**

**A B C D**

**E F G H**

**A B C D**

**E F G H**

**A B C D**

**E F G H**

**A B C D**

**E F G H**

**A B C D**

**E F G H**

**A B C D**

**E F G H**

**A B C D**

**E F G H**

**A B C D**

**E F G H**

**A B C D**

**E F G H**

**A B C D**

**E F G H**

**A B C D**

**E F G H**

**A B C D**

**E F G H**

**A B C D**

**E F G H**

**A B C D**

**E F G H**

**12**

**11**

**10**

**9**

**8**

**7**

**6**

**5**

**4**

**3**

**2**

**1**

**12**

**11**

**10**

**9**

**8**

**7**

**6**

**5**

**4**

**3**

**2**

**1**

**A B C D E F G H**

**III.2 Washing and developing slides using the colour precipitation method**

Remove chambers and put aside for washing before re-use. As the chambers are removed from each slide immerse the slide briefly in the first wash solution before placing them in the rack.

Place the rack containing the slides in the VP2000 processor and load the following washing programme:

15 min in 2 x SSC/50% formamide at 40°C with constant agitation

40 min in same at 50°C with constant agitation

20 min in 1 x SSC/50% formamide at 50°C with constant agitation

5 min in 1 x SSC at room temp. (agitation)

5 min in 1x TBS at room temp.

At this point the slides are removed from the VP2000 and placed in a flat tray, sections facing up.

BLOCK (1% Roche blocking agent in TBS) 1 h

1 x TBS containing 1/3000 dilution of Anti-DIG AP and 0.05% Tween-20 1h

Wash 4 times for 10 min each in 1 x TBS.

5 min in AP-Buffer

Develop in AP-Buffer containing 2 μl NBT/ml and 1.5 μl BCIP/ml

Since the post-hybridization treatments are more rapid than conventional protocols, it means that developing is commenced early in the day. Appearance of signals is monitored as soon as 15 min after addition of developer and expect to detect initial signals from highly expressed genes such as histone after this time. This can be used to gauge the general sensitivity of the *in situ* experiment.

Stop all reactions after 24 h: wash several times in water, then 70% ethanol and 100% to remove excess stain, but monitor carefully so that signal is not also diminished.

Allow to dry.

Add 2-3 drops of entellan and mount. Dry overnight in the fumehood and view.

### IV. Monitoring results and background signals

An initial scan of the results is performed by eye (or a dissecting microscope). The wheat grain are large enough to allow early indication of varied expression patterns: some genes expressed in maternal tissues at 3DAA show disproportionate signal above the crease region, genes expressed in dividing cells at 6DAA light up the periphery of the endosperm, genes localised to the modified aleurone are recognisable at 6 and 9 DAA and “conventional” bulk endosperm expressers are easily identified at 9 DAA. Though crude and biased towards strong signals, it provides indications as to whether the experiment was successful. With controls like Histone H4, Alpha-thionin and Poly(T)-DIG showing different spatial patterns of expression, it indicates that all cell types within the tissue sections are receptive to probes.


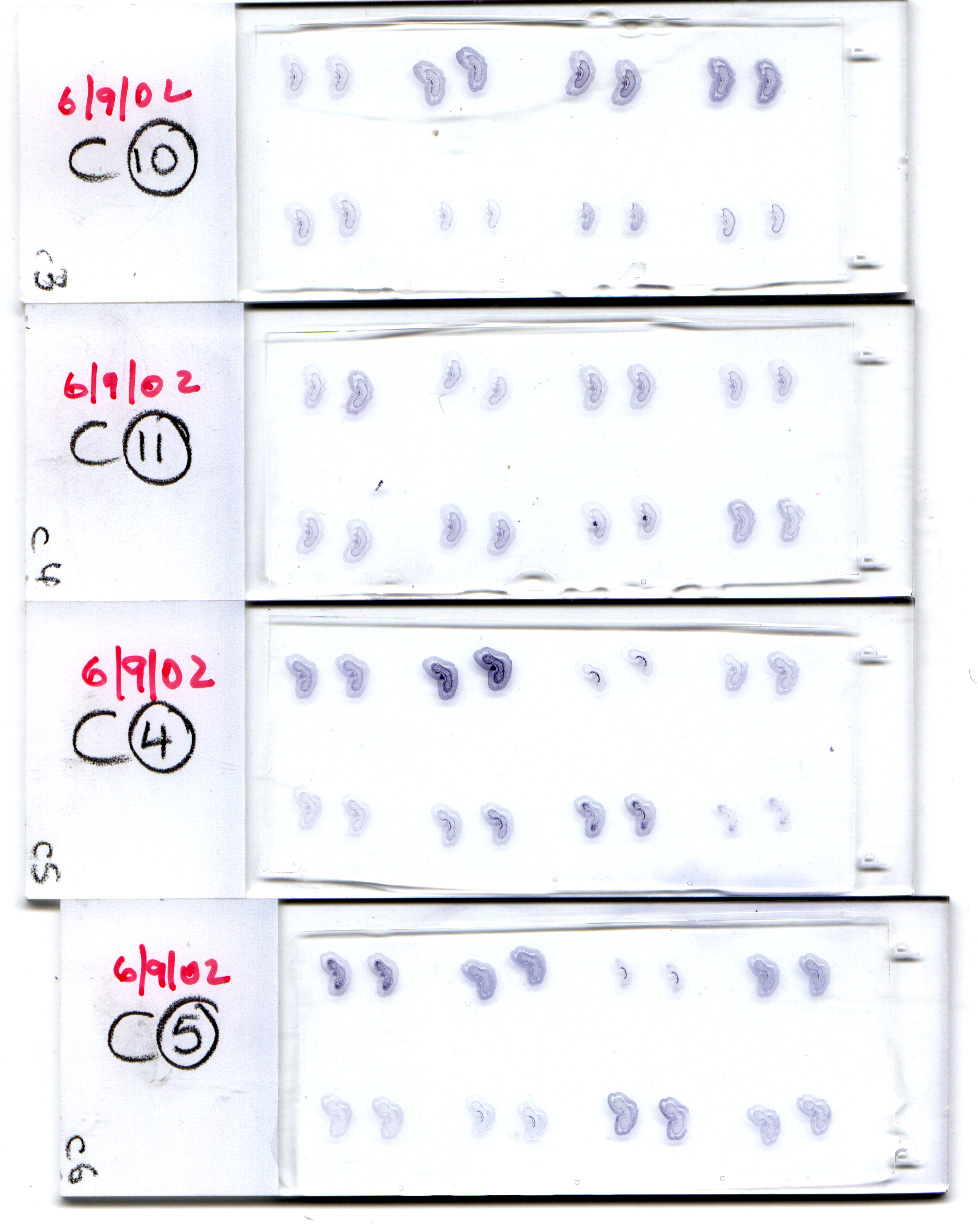

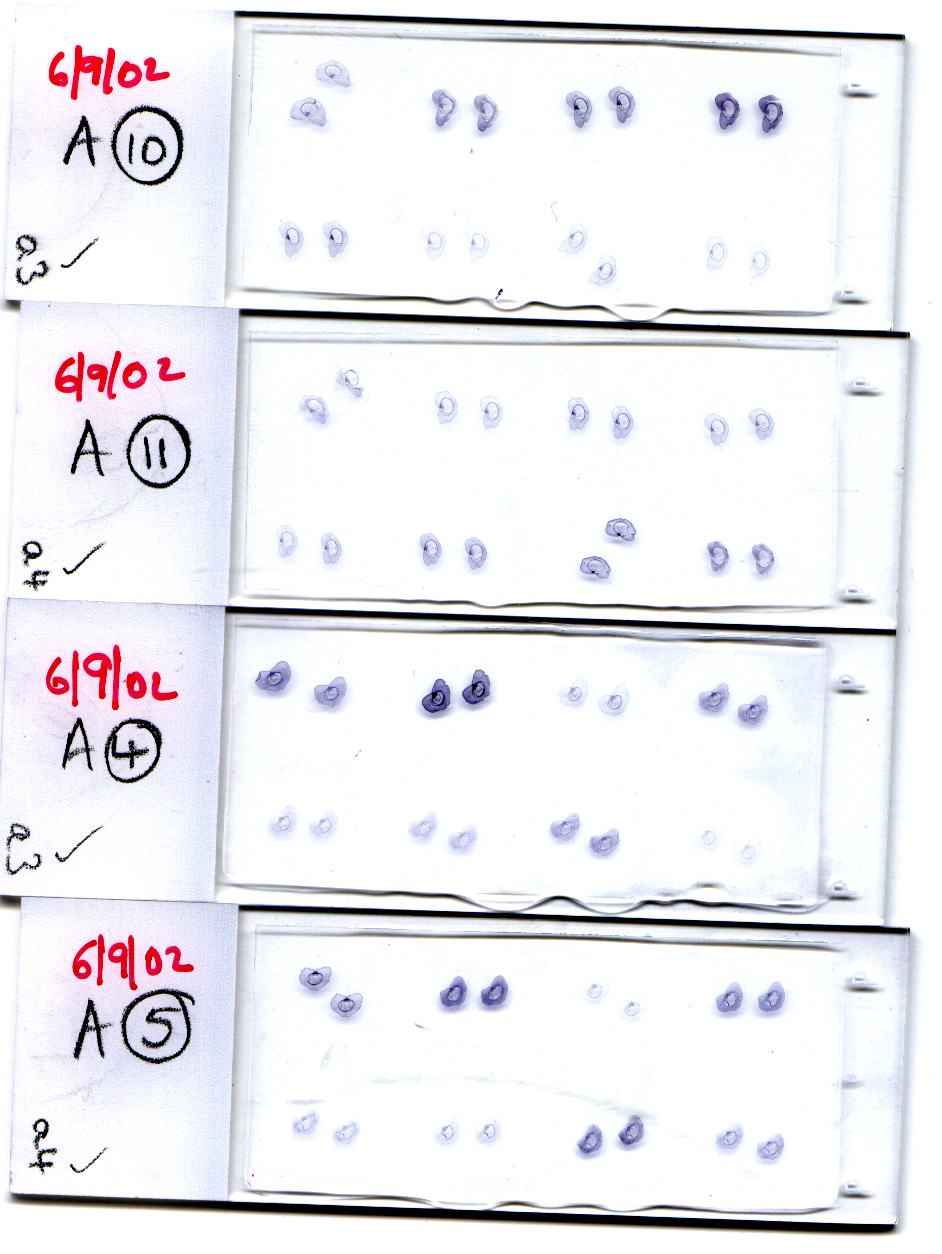


# Stage C = 9DAA

# Stage A = 3DAA

Slides **scanned** in.

One of the main problems impeding rapid evaluation of *in situ* output is the background-staining factor. It complicates the detection of a specific spatial pattern of colour distribution and mitigates confidence in any visible signal that may be detected. One of the most obvious determinants of background is the **amount of probe** used in the hybridisation. Also a factor is the **physical trapping** of probe/stain particularly at the junction of the different tissue types: pericarp to nucellus, nucellar projection to endosperm cavity etc. Finally, if the developing time is left to proceed for too long the background builds up.

For screening purposes it would be too time-consuming to individually optimise conditions for each probe and by stopping the developing reaction of all probes at 24 h, it is probably that this will compromise the detection of low abundance transcripts. In the context of large-scale screening, a certain amount of background can be accommodated and can indicate: a) the staining is working and b) an estimation of the ratio of background to other signal can be quite informative e.g. NO background and no signal could suggest repeating the *in situ* with more probe or longer development time. The advantage of using several probes is that each acts as a control for the other; background signal distributions are usually similar and anything that contrasts may indicate something interesting.

The poly(T)-DIG oligo control is included as a positive control to ascertain the distribution of the entire complement of messenger RNA. Patterns of colour distribution contrasting to this may be interesting while identical patterns can often be attributed to the probe concentration.

### V. Data handling

There will be a huge volume of information to record in some coherent and accessible format **throughout** the process, even prior to obtaining any pictures. Spreadsheets are probably the method of choice in terms of recording probe details etc.

Low magnification ( x 2) images of each section could be the default requirement. Anything of interest will be subject to subsequent more detailed screens producing more images. Also, the slides themselves will have to be stored and catalogued.
